# Extraordinary Delayed-Onset Negative Pressure Pulmonary Hemorrhage Resulting in Cardiac Arrest after General Anesthesia for Vocal Cord Polypectomy

**DOI:** 10.1155/2020/8830935

**Published:** 2020-11-17

**Authors:** Masahiro Koide, Tatsuya Kitada, Masaya Kogure, Kento Fukui, Koji Sogabe, Yukinori Kato, Hiroki Kitajima, Satoshi Akabame

**Affiliations:** Department of Cardiovascular Medicine, Kyoto Okamoto Memorial Hospital, Kyoto, Japan

## Abstract

Negative pressure pulmonary edema and hemorrhage are uncommon but potentially life-threatening complications associated with general anesthesia. Postoperative negative pressure pulmonary edema usually occurs immediately after surgery, and delayed-onset cases occurring more than 1 hour after surgery have rarely been reported. A 37-year-old woman with bronchial asthma underwent vocal cord polypectomy under general anesthesia in another hospital and experienced cardiac arrest due to a negative pressure pulmonary hemorrhage occurring 3 hours and 30 minutes after surgery. She was successfully treated with venoarterial extracorporeal membrane oxygenation and completely recovered without any complications. Extraordinary delayed-onset negative pressure pulmonary hemorrhage occurring more than three hours after surgery has rarely been reported. This case may indicate the need for more careful observation of patients following surgery.

## 1. Introduction

Negative pressure pulmonary edema (NPPE) is a well-recognized cause of acute respiratory failure that occurs after strong inspiratory efforts against upper airway obstruction [1]. NPPE could progress to diffuse alveolar hemorrhage (DAH), and NPPE-related DAH is rare but a significant life-threatening complication associated with general anesthesia [2]. Postoperative NPPE usually occurs immediately after surgery, and few late-onset cases occurring more than 1 hour after surgery have been reported [3–6]. We present a case of extraordinary delayed-onset negative pressure pulmonary hemorrhage resulting in cardiac arrest after general anesthesia for vocal cord polypectomy.

## 2. Case Presentation

A 37-year-old woman with a history of bronchial asthma underwent vocal cord polypectomy in another hospital. General anesthesia was induced with 0.5 *μ*g/kg/min of remifentanil and 100 mg of propofol following administration of 0.5 mg of atropine and 6.6 mg of dexamethasone, and 35 mg of rocuronium was administered for muscle relaxation. Tracheal intubation was easily conducted. Anesthesia was maintained with 1.5% of sevoflurane and 0.25 *μ*g/kg/min of remifentanil, and the surgery proceeded uneventfully. 200 mg of sugammadex sodium was used for muscle recovery, and extubation was performed uneventfully. The duration of anesthesia was 55 minutes and the operative time was 17 minutes. She was transferred to the recovery room, and 3 hours and 20 minutes after the operation, her condition was confirmed to be stable with 97% oxygen saturation under room air. However, 10 minutes later, she suddenly complained of nausea and sickness, followed by repeated severe and persistent vomiting. Eight minutes after her first complaint, her consciousness level was down, resulting in cardiopulmonary arrest. A monitor electrocardiogram revealed ventricular fibrillation, and defibrillation by an automated external defibrillator was performed five times and administration of adrenaline 1 mg was repeated three times in another hospital; however, return of spontaneous circulation (ROSC) was not obtained. Tracheal intubation was performed, but no obvious laryngeal edema or foreign bodies in the respiratory tract were observed.

**Figure 1:**
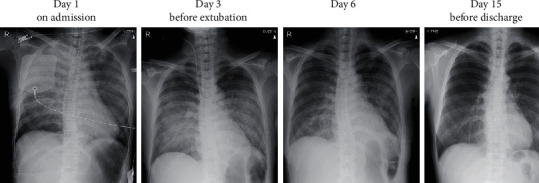
The course of chest X-ray images from admission to discharge. Images on admission (day 1) showed bilateral interstitial opacities, which gradually resolved during hospitalization.

**Figure 2:**
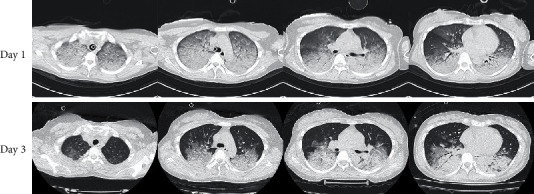
Chest computed tomography images on days 1 and 3 of admission. The scan on admission (day 1) showed bilateral pulmonary consolidations accompanied by air bronchogram and the ground-glass opacities. After extubation (day 3), the consolidations and opacities tended to improve but still remained.

**Figure 3:**
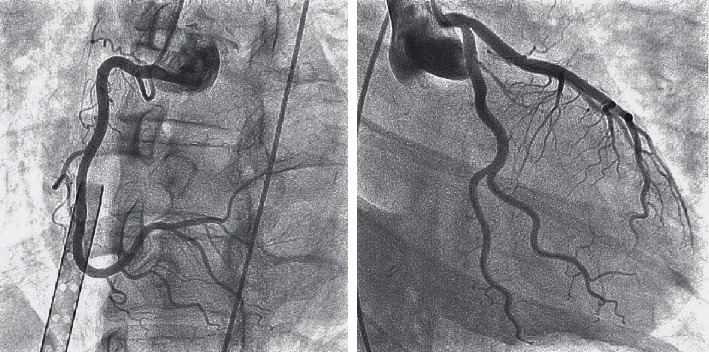
Coronary angiography showing no significant stenosis.

**Figure 4:**
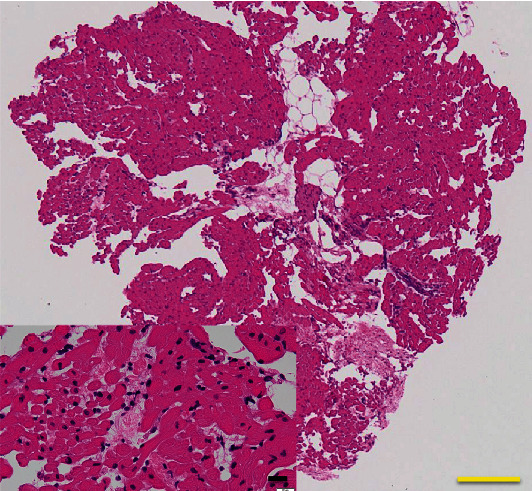
Histological images of biopsied myocardium. Yellow scale bar = 20*μ*m; black scale bar = 20*μ*m.

She was transferred to our hospital while cardiopulmonary resuscitation was continued. Although she was in cardiac arrest with pulseless electrical activity on arrival at our hospital, ROSC was obtained with administration of adrenaline 1 mg. The duration of cardiac arrest was estimated as 44-47 minutes. Arterial blood gas analysis revealed severe acidosis and hypoxemia, with a pH of 6.752, PaO2 of 69.5 mmHg, PaCO_2_ of 56.9 mmHg, base excess of -28 mmol/L, and lactate of 18.6 mmol/L. The electrocardiogram showed no ST elevation. Chest X-ray revealed bilateral interstitial opacities (Figure 1). Transthoracic echocardiography showed severe left ventricular systolic dysfunction with eyeballing of left ventricular ejection fraction of 20%. Chest computed tomography scan showed bilateral pulmonary consolidation accompanied by air bronchogram and the ground-glass opacities (Figure 2). Venoarterial extracorporeal membrane oxygenation (VA-ECMO) was applied because of hemodynamic instability and advanced hypoxemia, even with administration of 100% oxygen by a ventilator. Coronary angiography showed no significant stenosis in the coronary arteries (Figure 3). A myocardial biopsy was performed from the endocardium of the right ventricular septum, but later pathological analysis showed no abnormalities of the myocardium and no evidence of myocarditis (Figure 4).

The patient was returned to the intensive care unit. A large amount of hemoptysis, estimated to be about 200 mL within 12 hours, was drained from the tracheal tube. On day 2 after admission, she was weaned from the VA-ECMO. On day 4 of admission, she was extubated with confirmation of clear consciousness. Although her oxygenation recovered rapidly to PaO2 of 86 mmHg under an administration of 3 L/min via nasal cannula, hemoptysis persisted after extubation. The chest computed tomography scan still showed diffuse bilateral ground-glass opacities and partial consolidation (Figure 2). Transthoracic echocardiography showed rapid recovery of left ventricular function, and right heart catheterization showed normal hemodynamics (pulmonary capillary wedge pressure 2 mmHg), indicating that the cause of pulmonary consolidation was not cardiogenic pulmonary edema but pulmonary alveolar hemorrhage. Immunological tests such as antiglomerular basement membrane antibodies, antinuclear factors, antineutrophil cytoplasmic antibodies (ANCA), anti-*β*2-glycoprotein 1 antibodies, anti-cardiolipin antibodies, and lupus anticoagulant were negative. No coagulation abnormalities could be confirmed. These results ruled out the complications of underlying diseases causing pulmonary alveolar hemorrhage, including ANCA-associated vasculitis, antiphospholipid antibody syndrome, Goodpasture's syndrome, or abnormal coagulation. Coronary artery spasm was not induced during a provocation test by intracoronary administration of acetylcholine, and critical ventricular arrhythmia was not induced during electrophysiological study.

**Figure 5:**
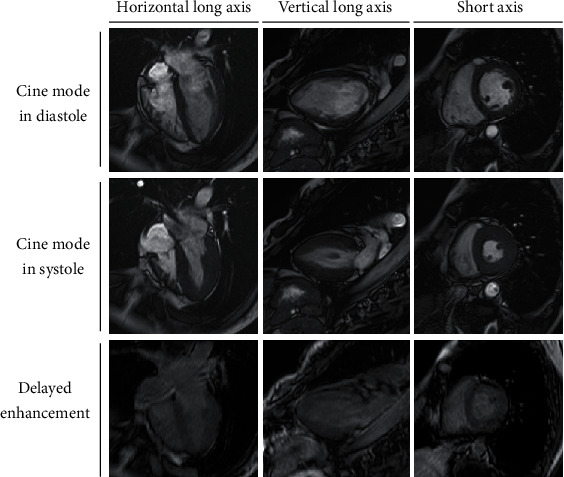
Cardiac magnetic resonance imaging showing no findings of morphological abnormalities or delayed enhancement.

The patient was discharged after 15 days in the hospital without any neurological complications. Cardiac magnetic resonance imaging showed no findings of morphological abnormalities or delayed enhancement (Figure 5).

## 3. Discussion

NPPE and hemorrhage, which are caused by loading excessive intrathoracic negative pressure due to inspiration effort against obstruction of the upper airway, are uncommon but potentially life-threatening complications associated with general anesthesia involving tracheal intubation [1, 2]. The causes of total upper airway obstruction occurring after extubation after general anesthesia are laryngeal edema, laryngospasm, and aspiration of foreign bodies [7–9]. In this case, there was no mechanical obstruction in the upper respiratory tract during reintubation performed for cardiac arrest, which suggested that the cause of upper airway obstruction was laryngeal spasm. Laryngeal spasm is a spontaneous, irreversible reflex closure of the glottis caused by intrinsic or extrinsic laryngeal stimulation [10]. Postoperative laryngeal spasm, with an incidence of 0.01%-0.05% of all anesthetic procedures, is the most common cause of NPPE in adults [2, 11–13], and NPPE may occur in up to 4% of cases of laryngeal spasm [2, 7]. Postoperative laryngeal spasm is usually induced by airway manipulation, blood and secretions in the pharynx, or vomiting [7]. Although postoperative NPPE usually occurs immediately after extubation, some delayed-onset cases occurring from 1 hour to 14 hours after surgery have been reported [3–6]. In the present case, we considered that severe persistent vomiting that occurred more than 3 hours after surgery caused laryngospasm that resulted in NPPE.

In the treatment of NPPE, sufficient oxygenation or noninvasive positive pressure ventilation is effective, and reintubation is sometimes necessary in severe cases; however, cases requiring extracorporeal membrane oxygenation have rarely been reported [14–16]. The cause of postoperative pulmonary edema is either cardiogenic or noncardiogenic [17]. In the present case, because transthoracic echocardiography after ROSC showed severe left ventricular systolic dysfunction, we initially assumed that the patient had some heart disease that caused acute heart failure resulting in cardiac arrest. However, rapid recovery of cardiac function, absence of abnormal findings in myocardial histology and cardiac magnetic imaging, and normal pulmonary capillary wedge pressure in spite of persistent hemoptysis were observed, which indicated that the left ventricular systolic dysfunction noted after ROSC was caused by myocardial stunning after resuscitation [18, 19].

## 4. Conclusions

We present a case of extraordinary delayed-onset negative pressure pulmonary hemorrhage (NPPH) resulting in cardiac arrest after general anesthesia for vocal cord polypectomy. The patient was successfully treated with VA-ECMO and completely recovered without complications. NPPH occurring more than 3 hours after surgery has rarely been reported. This case may indicate the need for more careful observation of patients after surgery.
